# Fibromyalgia is linked to increased subjective sensory sensitivity across multiple senses

**DOI:** 10.1177/03010066241234037

**Published:** 2024-02-26

**Authors:** Chloe Rafferty, Jamie Ward

**Affiliations:** 1948 University of Sussex, UK

**Keywords:** pain, disorders, individual differences, fibromyalgia, sensory sensitivity

## Abstract

Changes in subjective sensory sensitivity – reporting sensory stimuli as being atypically intense or weak – are a transdiagnostic symptom of several disorders. The present study documents for the first time the sensory sensitivity profile of fibromyalgia, taking a questionnaire measure that asks about different sensory modalities and both hyper- and hyposensitivity (the Glasgow Sensory Questionnaire, GSQ). The fibromyalgia group had higher overall scores on this measure. This was linked more strongly to sensory hypersensitivity and was pervasive across all senses that were surveyed. Although differences in hyposensitivity were found, these were sporadic (perhaps linked to the symptoms of fibromyalgia itself) and did not resemble the pattern documented for autism (e.g., self-stimulating and repetitive behaviours were not a feature of fibromyalgia). We suggest that individual differences in subjective sensory hypersensitivity may be a multisensory dispositional trait linked to fibromyalgia which ultimately becomes most pronounced for pain.

Fibromyalgia is a chronic condition characterised by widespread pain, fatigue, and cognitive dysfunction, such as forgetfulness (Häuser et al., 2015). Despite the trials of multiple diagnostic criteria (Bennett, 1981; Wolfe, 1986), widespread pain and tender points persist as trademarks for diagnosing fibromyalgia. The 1990 criteria from the American College of Rheumatology (ACR) gained official recognition for fibromyalgia in the International Classification of Diseases (ICD) in 1992, due to its emphasis on widespread pain (Wolfe et al., 1990). Fibromyalgia is now recognised as a disease by the World Health Organization (WHO) due to the work of the 1990 ACR diagnostic criteria (Galvez-Sánchez & Reyes del Paso, 2020). The prevalence of fibromyalgia is approximately 3–6% of the general population (Sarzi-Puttini et al., 2020; Vincent et al., 2013) and is especially common in women, with an estimated 2:1 ratio (Clauw, 2014).

Since the initial diagnostic criteria, several changes have been made, and the currently acknowledged 2010 criteria utilises the Widespread Pain Index (WPI) and the Symptom Severity (SS) scale (Wolfe et al., 2010). There have been multiple modification proposals for the diagnostic criteria over the years, but none have been approved by the ACR yet (Galvez-Sánchez & Reyes del Paso, 2020). Notably, the conceptualisation of the condition has been raised since the 2010 criteria to classify fibromyalgia as more multisymptomatic than simply painful bodily sensations (Jones et al., 2015). Chronic fatigue is also a pervasive symptom in fibromyalgia, affecting up to 81% of sufferers (Guymer & Clauw, 2002) and largely contributing to life impairment (Wolfe et al., 1990, 1996). Up to 75% of individuals with fibromyalgia express concerns of such cognitive problems, including concentration and memory (Bell et al., 2018). One study found that some fibromyalgia patients display the memory of someone approximately 20 years their elder (Glass, 2008).

The present study addresses the possibility that fibromyalgia stems, at least in part, from altered central (i.e., brain-based) sensory processing mechanisms. These may include, but are not limited to, pain. Evidence for this comes from studies showing lower pain thresholds in neutral parts of the body (i.e., that are not linked to symptomatic pain), such as pressure applied to the thumb (Geisser et al., 2008). Similarly, individuals with fibromyalgia also report lower thresholds of pain towards auditory stimuli (Staud et al., 2021) and increased visual discomfort when viewing certain high spatial frequency patterns (Ten Brink & Bultitude, 2022). The latter is known to be of early visual cortical origin (O'Hare & Hibbard, 2016), although additional types of visual disturbance possibly originating from the eye have also been reported (Zdebik et al., 2021). Finally, vestibular complaints such as dizziness is a common complaint in fibromyalgia (Bayazıt et al., 2002; Clauw, 1995; Peinado-Rubia et al., 2020). In sum, there is good indicative evidence for wider-ranging differences in sensory sensitivity in fibromyalgia beyond the defining characteristic of localised pain and tenderness.

Ward (2019) distinguishes between usages of the term ‘sensory sensitivity’ and discusses how they may be mapped to different mechanisms. Subjective sensory sensitivity refers to differences in self-reported intensity such as those measures by discomfort thresholds (and considered in the previous paragraph). Neural sensory sensitivity refers to interindividual differences in the extent to which a given sensory stimulus tends to elicit a large or small neural responses (e.g., as measured in fMRI, EEG, or intracranially). There is an assumed monotonic relationship between subjective sensory sensitivity and neural sensory sensitivity, although how and where it is instantiated in the brain (e.g., early sensory cortices, or affective hubs such as the insular cortex) remains to be elucidated (López-Solà et al., 2014, 2017). Increased subjective and neural sensory sensitivity could arise either from increased stimulus-relevant activity (i.e., signal gain) or stimulus-irrelevant activity (i.e., enhanced noise), in which it is impossible a priori to link this to the third concept, namely, behavioural sensory sensitivity (i.e., the detection and discrimination of stimuli). The current study is concerned with subjective sensory sensitivity, as measured via questionnaire, although it is to be noted that other studies have looked at behavioural sensory sensitivity. For example, fibromyalgia is linked to poorer performance on detection and discrimination of tastes and smells (Amital et al., 2014; Blanco et al., 2018; Özsoy-Ünübol et al., 2020), but it is unknown whether this is accompanied by higher subjective sensory sensitivity (i.e., finding smells and tastes unusually intense or aversive).

Subjective sensory sensitivity is measured here using the Glasgow Sensory Questionnaire (GSQ; Robertson & Simmons, 2013) which contains equal numbers of questions about seven modalities: vision, audition, tactile, olfactory, gustatory, vestibular, and proprioception. Questions are also equally divided between hyper- and hyposensitivity. The measure was developed from reports of sensory sensitivity of autism but are entirely generic in nature (none refer to autism explicitly) and can be construed as a transdiagnostic measure that has been psychometrically validated in the nonclinical general population across several languages (Kuiper et al., 2018; Robertson & Simmons, 2013; Sapey-Triomphe et al., 2017; Takayama et al., 2014). Questions tend to load on a single factor or possibly two factors representing hyper- and hyposensitivity but noting that these two factors tend to positively correlate (one possible explanation for this is that some people have more variable sensory responses that can be extreme in either direction; see Ward, 2019). The concept of hypersensitivity is more directly relevant to fibromyalgia, but all questions are included for completeness. It is to be noted that hyposensitivity is not necessarily the opposite of hypersensitivity and includes concepts such as self-stimulation (e.g., repetitive behaviours in a given sensory domain) which could reflect weaker sensory experiences but need not (see Ward, 2019, for discussion). Our approach of asking about multiple senses, excluding pain, is similar to that of a recent research by Wang et al. (2022, 2020) who developed their own multisensory measure.

Two hypotheses can be generated for the present study: **Hypothesis 1**: It is assumed that the fibromyalgia group will present greater overall GSQ scores compared to the control group. **Hypothesis 2**: It is expected that the group with a fibromyalgia diagnosis will report higher levels of hypersensitivity in comparison to the control group across multiple senses.

## Methods

### Participants

There were 359 participants included after removal of incomplete responses in two groups: individuals who have been medically diagnosed with fibromyalgia (*N* = 299; mean age = 45.72 years, *SD* = 10.45, range = 18–75; 10 men, 288 women, 1 nonbinary) and a control group who have not been diagnosed with the condition (*N* = 60; mean age = 31.15 years, *SD* = 15.38, range = 18–73; 11 men, 49 women). Although the groups are not matched demographically (age: t(70.319) = 7.019, *p* < .001; gender: χ^2^(1) = 20.290, *p* < .001), we show below that age and gender are unrelated to GSQ scores, as also shown previously (Robertson & Simmons, 2013). A sensitivity analysis, conducted on G*Power, indicated that this sample size was sufficient to detect group differences of Cohen's *d* of 0.35 and above (at power = 0.8, alpha = 0.05); thus, the design was sensitive to small effects. Participants with fibromyalgia were largely collected from the UK fibromyalgia support group (via their Facebook page), and controls were recruited via email and social media.

Ethical approval was obtained from the School of Psychology at the University of Sussex.

### Materials and Procedure

Participants completed this survey remotely using Qualtrics. After taking consent, participants supplied demographic information and answered the question ‘Have you been diagnosed with fibromyalgia?’ with options ‘yes’ or ‘no’. This was used to assign groups.

The GSQ consists of 42 Likert scale questions and participants indicated their level of agreement with statements on a 5-point scale ranging from ‘*Never*’, ‘Rarely’, ‘Sometimes’, ‘Often’ and ‘*Always*’ and responses are coded on a scale from 0 to 4. An example item is ‘Do bright lights ever hurt your eyes/cause a headache?’. The survey took around 10–15 min to complete. Cronbach's alpha for the GSQ for our sample was 0.74, which implies acceptable reliability.

## Results

**Figure 1.**
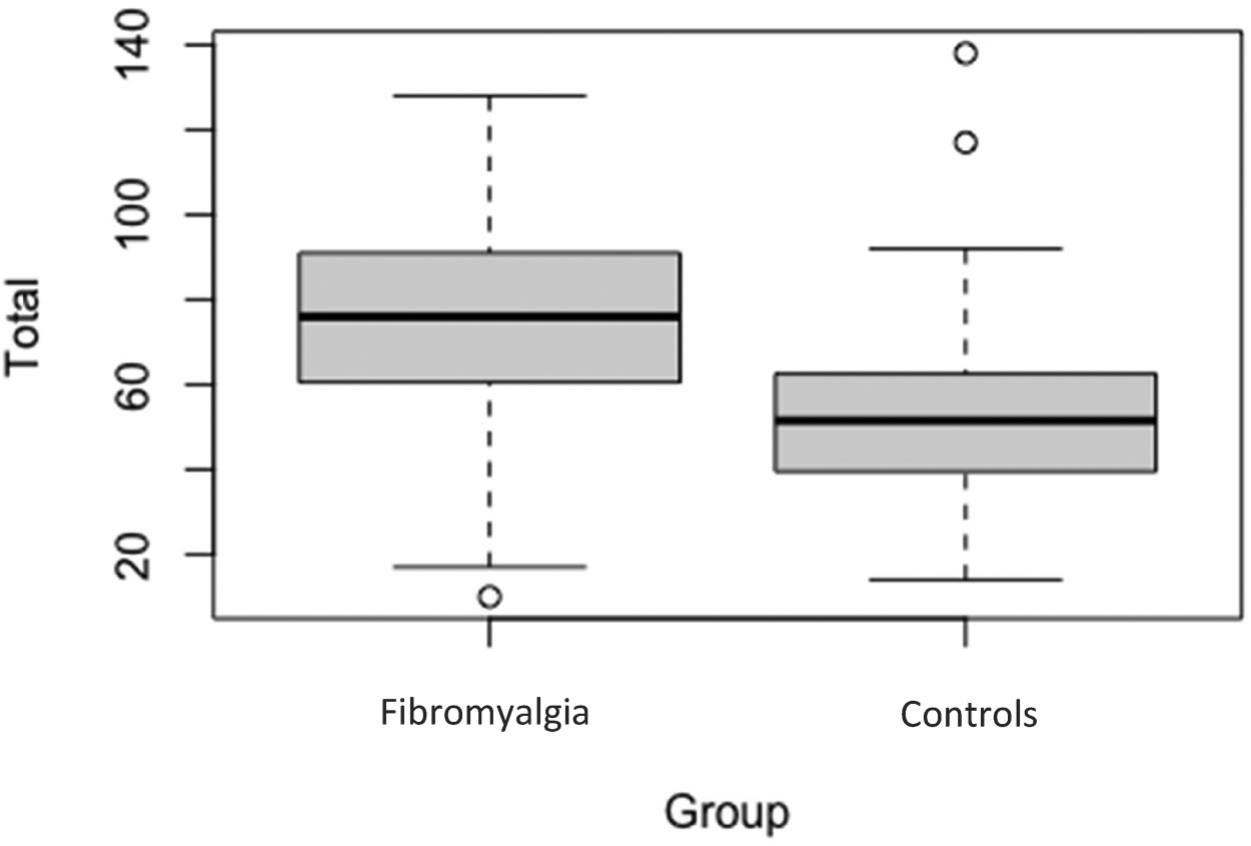
Total mean GSQ scores between groups. Bold lines represent the mean score for each group. Shaded regions represent the lower and upper quartiles. Error bars represent standard error.

First of all, we establish that GSQ scores are not significantly associated with age (fibromyalgia: *r* = −.085, controls *r* = −.218, *p’s* > .05) or male versus female gender (fibromyalgia: t(296) =  −1.120, controls t(58) =  −0.271, *p’s* > .05). These demographic variables are collapsed for further analyses. Figure 1 shows the mean and distribution of overall scores on the GSQ. The fibromyalgia group had a mean score of 75.59 (*SD* = 21.82) compared to the control mean of 53.30 (*SD* = 22.37), and this difference was significant (t(357) = 7.19, *p* < .001) with a large effect size (Cohen's *d* = 1.02).

**Figure 2.**
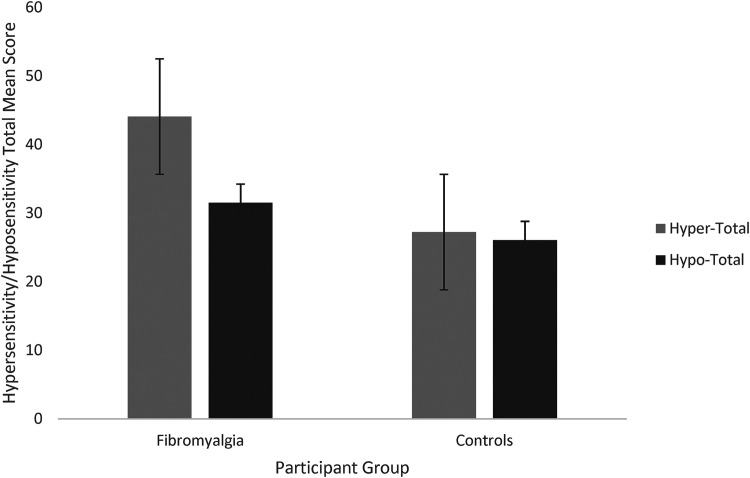
Total mean hypersensitivity and hyposensitivity scores between groups. Note: Error bars represent standard error.

Figure 2 shows the GSQ questions broken down into hypersensitivity and hyposensitivity. A two-way mixed ANOVA revealed a main effect of group (F(1, 357) = 51.69, *p* < .001, η^2^ = 0.126), a main effect of hyper- versus hyposensitivity (F(1, 357) = 128.71, *p* < .001, η^2^ = 0.265), and an interaction between the two (F(1, 357) = 88.670, *p* < .001, η^2^* *= 0.199). Thus, fibromyalgia is linked to both increased multisensory hypersensitivity (as hypothesised; t(357) = 9.14, *p* < .001, *d* = 1.29) and hyposensitivity (where we had no initial hypothesis; t(357) = 3.724, *p* < .001, *d* = 0.53), but with a far larger group difference for hypersensitivity (as shown by the interaction).

**Figure 3.**
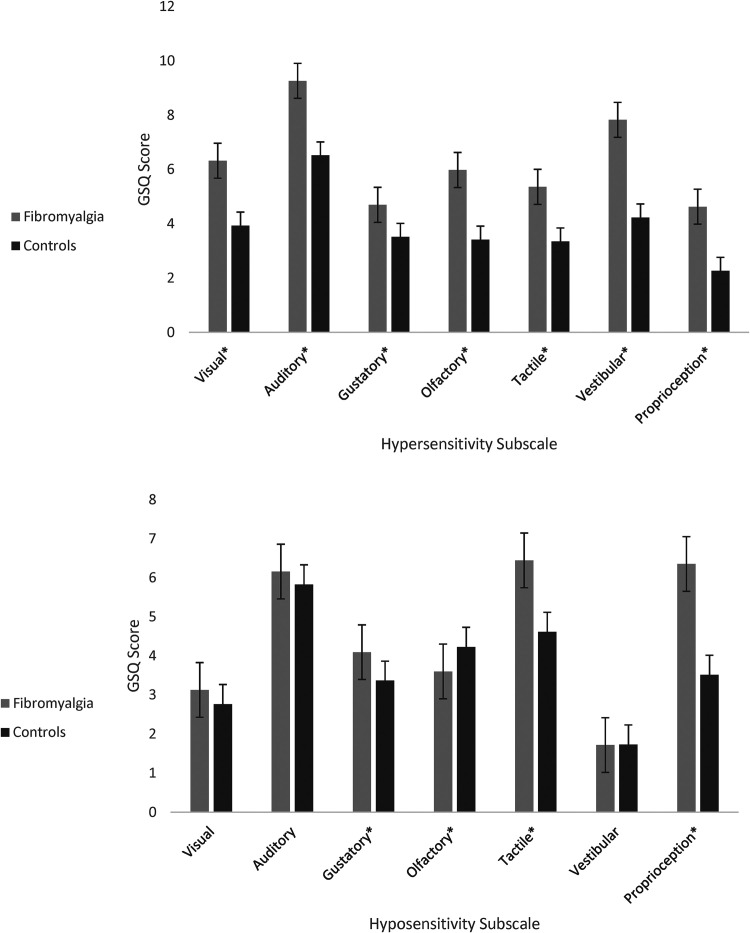
Mean GSQ hypersensitivity scores (top) and hyposensitivity scores (bottom) between groups. Note: Error bars represent one standard error. * *p* < .05.

Figure 3 shows the hypersensitivity and hyposensitivity scores broken down by sensory modality. A 2 × 7 ANOVA for hypersensitivity shows a main effect of group (F(1, 357) = 83.48, *p* < .001, η^2^ = 0.190), a main effect of sensory modality (F(1, 357) = 124.35, *p* < .001, η^2^ = 0.258), and a significant interaction (F(1, 357) = 7.64, *p* < .001, η^2^ = 0.021). Similarly, a 2 × 7 ANOVA for hyposensitivity shows a main effect of group (F(1, 357) = 13.87, *p* < .001, η^2^ = 0.037), a main effect of sensory modality (F(1, 357) = 130.46, *p* < .001, η^2^ = 0.268), and a significant interaction (F(1, 357) = 20.171, *p* < .001, η^2^ = 0.053). Post-hoc t-tests were used to clarify which senses were more implicated. Group differences were pervasive across all hypersensitivity domains (*p* < .001, all below a Bonferroni correction of .05/7), but for hyposensitivity they were found for four modalities with the body senses of touch and proprioception being the only ones to survive Bonferroni correction (*p* < .05/7). These may be a more direct reflection of fibromyalgia itself (e.g., including reports of numbness and problems with manual dexterity). Table 1 shows the full list of items from the GSQ ranked by Cohen's *d* effect size.

**Table 1.** All 42 individual items from the GSQ ranked (high to low) for Cohen's *d* effect size for fibromyalgia > controls. Note that *d* > .8 is considered large, *d* > .5 is medium, and *d* > .2 is small. Parentheses show the sensory domain and whether hyper- or hyposensitive.

Question	Cohen’s *d*
Do you feel ill/dizzy/peculiar if you have to reach up high or bend down low for something? (hyper-vestibular)	1.222
Do you find it difficult to tie your shoelaces or button up your clothes? (hyper-proprioception)	1.177
Do you find certain noises/pitches of sound annoying? (hyper-auditory)	1.092
Does your body ever feel ‘numb’ – like you cannot feel anything against your skin? (hypo-tactile)	0.998
Do you find it difficult to manipulate your hands when completing a delicate task (for example, picking up small objects or transferring objects from one hand to the other)? (hypo-proprioception)	0.994
Do bright lights ever hurt your eyes/cause a headache? (hyper-visual)	0.988
Do you dislike walking on uneven surfaces? (hyper-vestibular)	0.982
Do you dislike loud noises? (hyper-auditory)	0.965
Do you find it difficult to go into a strong-smelling shop (e.g., ‘Lush’ and ‘The BodyShop’)? (hyper-olfactory)	0.881
Do lights ever seem to flicker when you look at them? (‘Flickering’ in this question means appearing to turn on and off very quickly instead of appearing constant). (hyper-visual)	0.810
Do you react very strongly when you hear an unexpected sound? (hyper-auditory)	0.778
Do you think you have a weak sense of taste? One example of this would be if most food taste of ‘nothing’? (hypo-gustatory)	0.771
Do you stand very close (e.g., less than 1 m/3 ft away) or very far (e.g., more than 3 m/9 ft away) when you are talking to someone? (hypo-proprioception)	0.740
Do you ever feel ill just from smelling a certain odour? (hyper-olfactory)	0.727
Do you find it difficult to hear what people are saying? (hypo-auditory)	0.666
Do you ever feel dizzy/ill when playing fast-paced sports, for example, basketball or football? (hyper-vestibular)	0.664
Do you dislike having a haircut (e.g., because little bits of hair go down your back)? (hyper-tactile)	0.635
Do you dislike the physical sensation you get when people hug you? (hyper-tactile)	0.571
Do you find that you are unaware of your body's signals (e.g., do not often feel hungry/tired/thirsty)? (hypo-proprioception)	0.559
Do you gag when you are eating certain foods, perhaps feeling as if you are going to be sick? (hyper-gustatory)	0.528
Do you avoid going to restaurants because you can smell a certain odour? (hyper-olfactory)	0.482
Do you eat the same foods most of the time? (hypo-gustatory)	0.379
Do you rock yourself backwards and forwards? (hypo-vestibular)	0.377
Do you find that you position your body in a way that is different to most people (e.g., lie on your back on a sofa with your legs straight up in the air at a 90° angle)? (hyper-proprioception)	0.356
Do you find yourself fascinated by small particles (e.g., little ‘bits’ of dust in the air)? (hyper-visual)	0.340
Do you ever run your hand around the outside of an object before picking it up? (hypo-proprioception)	0.320
Do you cut the labels out of your clothes? (hyper-tactile)	0.304
Do you like to wear something/hold something (e.g., a hat or a pencil) so that you know where your body ‘ends’? (hyper-proprioception)	0.301
Do you use the tip of your tongue to taste your food before eating it? (hyper-gustatory)	0.283
Do you flick your fingers in front of your eyes? (hypo-visual)	0.278
Do you notice that you have hurt yourself but did not feel any pain? (hypo-tactile)	0.244
Do you hate the feel or texture of certain foods in your mouth? (hyper-gustatory)	0.225
Do you find that you are able to go outside without a coat or a jacket when other people think that it is too cold? (hypo-gustatory)	0.116
Do you smell your food before you eat it? (hypo-olfactory)	0.044
Do you like to listen to the same piece of music/part of a DVD over and over again? (hypo-auditory)	0.032
Do you like lining objects up? (hypo-proprioception)	−0.143
Are you ever told by others that you wear too much perfume/after-shave? (hypo-olfactory)	−0.147
Do you like to spin yourself round and round? (hypo-vestibular)	−0.244
Do you chew and lick objects that are not food (e.g., pen lids or bottle tops) because you like the way they feel in your mouth? (hypo-gustatory)	−0.304
Do you really like listening to certain sounds (e.g., the sound of paper rustling)? (hypo-auditory)	−0.306
Do you enjoy wearing very strong perfumes/after-shaves? (hypo-olfactory)	−0.457
Do you like to run about – perhaps up and down in straight lines or round in circles? (hypo-vestibular)	−0.586

## Discussion

This study shows that individuals with fibromyalgia experience greater levels of subjective sensory hypersensitivity across all senses that were questioned, not including any questions about pain which are part of the diagnostic criteria for this disorder. Chronic pain conditions such as fibromyalgia are often explained in terms of a central (i.e., brain-based) sensitisation to pain. The present research, alongside related studies (Harte et al., 2018; Wang et al., 2022), suggests that pain central sensitisation may need to be reconceptualised in terms of generalised multisensory central sensitivity or, relatedly, that generalised multisensory central sensitivity is a predisposing risk factor for the development of a more pain-specific profile. The extent to which sensory hypersensitivity is directly related to pain symptomatology or orthogonal to it is an important consideration for future research (e.g., Schrepf et al., , 2018, 2023). Wang and Frey-Law (2023) suggest that multisensory sensitivity may relate to the pain phenotype (fibromyalgia vs. low back pain) rather than pain presence per se (but see Bar-Shalita & Cermak, 2020). One explanation of this dissociation is that multisensory sensitivity is indeed a precursor to the development of certain forms of pain rather than a consequence of pain. Therefore, it could conceivably be utilised as a differential diagnostic measure. The absence of a measure of pain in the present study should be acknowledged as a limitation.

The present research used an existing questionnaire, the GSQ (Robertson & Simmons, 2013), and its use in the present context could be viewed as both a strength and a weakness. As a strength, this measure encompasses a wide variety of senses (e.g., the multisensory scale of Wang et al., 2020, did not include vestibular) and has been psychometrically validated in nonclinical samples. The fact that it was derived from reports of sensory sensitivity in autism could be viewed as either a strength (if one takes a transdiagnostic approach to such symptoms) or a weakness (if one assumes that fibromyalgia is special). Indeed, our results show an interesting mix of similarities and differences to that found in autism. The similarities include hypersensitivity to a wide range of everyday stimuli such as sounds, light, and touch. The latter even extends to a dislike of having a haircut (because of bits of hair against the skin): the effect size for fibromyalgia for this item was *d* = 0.64 (a medium effect). However, there were also a set of questions (see Table 1) where individuals with fibromyalgia tended to score lower than controls (negative effect sizes). These were related to repetitive and/or self-stimulating behaviours (spinning around, lining up objects, wearing strong scents) which are positively associated with autistic traits. Other ‘hyposensitivity’ questions that were positively linked to fibromyalgia could be construed as relating directly to this order (e.g., skin numbness, manual dexterity) rather than as being part of a wider multisensory hyposensitivity profile. Since conducting this research, another study applied an alternative measure of sensory sensitivity derived from the autism literature (Tavassoli et al., 2014) to a fibromyalgia group, also noting hypersensitivity in touch, smell, and vision but not for taste or hearing (Dorris et al., 2022).

Our research suggests that subjective sensory hypersensitivity extending across multiple senses is a key feature of fibromyalgia. It may also be a common transdiagnostic symptom for a range of other disorders, although further research is needed to establish whether superficial similarity (from questionnaires) maps on to any deeper similarity mechanistically (in terms of brain and behaviour). The current research is also necessarily correlational in nature and does not determine whether sensory hypersensitivity is a cause or consequence of fibromyalgia. Future research could either explore this longitudinally or determine whether sensory hypersensitivity acts as an endophenotype in nonaffected relatives of people with fibromyalgia (which would support the idea that it functions more as a risk factor).

The current data is inconsistent with the notion that sensory hypersensitivity stems from the sense organs themselves because it is unclear how they could all be coincidentally affected. Of course, one could potentially extend that logic to the brain itself given that early sensory cortices are largely modular in function. One possibility is that sensory hypersensitivity in the brain reflects hub regions of the brain, such as insula (López-Solà et al., 2014, 2017), which receive multiple sensory signals and may inappropriately amplify them (by tagging them as salient). However, the fact that sensory hypersensitivity reflects stimulus properties (e.g., spatial frequency in the case of vision) argues in favour of at least some role for sensory cortices and the existence of a generic brain-based mechanism that spans multiple sensory systems such as increased neural noise or the inability to predict and hence adapt to sensory stimuli (Ward, 2019).

## Supplemental Material

sj-xlsx-1-pec-10.1177_03010066241234037 - Supplemental material for Fibromyalgia is linked to increased subjective sensory sensitivity across multiple senses Supplemental material, sj-xlsx-1-pec-10.1177_03010066241234037 for Fibromyalgia is linked to increased subjective sensory sensitivity across multiple senses by Chloe Rafferty and Jamie Ward in Perception
